# Adipocytes promote cholangiocarcinoma metastasis through fatty acid binding protein 4

**DOI:** 10.1186/s13046-017-0641-y

**Published:** 2017-12-13

**Authors:** Jihua Nie, Jingying Zhang, Lili Wang, Lunjie Lu, Qian Yuan, Fangmei An, Shuyu Zhang, Yang Jiao

**Affiliations:** 1 Department of Radiation Genetics, School of Radiation Medicine and Protection, Medical College of Soochow University, Suzhou, 215123 China; 2 Collaborative Innovation Center of Radiological Medicine of Jiangsu Higher Education Institutions, Suzhou, 215123 China; 3 Department of Toxicology, School of Public Health, Medical College of Soochow University, Suzhou, 215123 China; 4 Department of Radiotherapy, First Affiliate Hospital of Soochow University, Suzhou, 215123 China; 5 Department of Gastroenterology, Wuxi People’s Hospital Affiliated to Nanjing Medical University, Wuxi, 214002 China

**Keywords:** Cholangiocarcinoma, Metastasis, Adipocytes, Fatty acid-binding protein 4 (FABP4), Epithelial-mesenchymal transition (EMT)

## Abstract

**Background:**

The early occurrence regional nodal and distant metastases cholangiocarcinoma (CCA) is one of the major reasons for its poor prognosis. However, the related mechanisms are largely elusive. Recently, increasing evidences indicate that adipocytes might be involved in the proliferation, homing, migration and invasion of several malignancies. In the present study, we attempt to determine the effects and possible mechanisms of adipocytes on regulating progression of CCA.

**Methods:**

Adipocyte–CCA cell co-culture system and CCA metastasis mice model were used to determine the effects of adipocytes on CCA metastasis. We identified the biological functions and possible mechanisms of adipocyte-derived fatty acid binding protein 4 (FABP4) in regulating the adipocyte-induced CCA metastasis and epithelial-mesenchymal transition (EMT) phenotypes, both in vitro and in vivo.

**Results:**

Adipocyte–CCA cell co-culture promotes the in vitro and in vivo tumor metastasis, leading to increased adipocyte-derived fatty acid absorbance and intracellular lipids of CCA cells, which indicates adipocytes might function as the energy source for CCA progression by providing free fatty acids. Further, highly expressed FABP4 protein was identified in adipose tissues and fully differentiated adipocytes, and upregulated FABP4 was also detected by qRT-PCR assay in CCA cells co-cultivated with adipose extracts as compared to parental CCA cells. The specific FABP4 inhibitor BMS309403 significantly impaired adipocyte-induced CCA metastasis and EMT phenotypes both in vitro and in vivo.

**Conclusions:**

Together, the results demonstrate that the adipocyte-CCA interaction and the energy extraction of CCA cells from adipocytes are crucial for the invasion, migration and EMT of CCA cells. FABP4 from adipocytes mediates these adipocyte-induced variations in CCA cells, which could serve as a potential target for the treatment of CCA.

**Electronic supplementary material:**

The online version of this article (10.1186/s13046-017-0641-y) contains supplementary material, which is available to authorized users.

## Background

Cholangiocarcinoma (CCA), which can be categorized into perihilar, distal and intrahepatic CCA, originates from epithelial cells located within the biliary tree [1]. In the United States, the cumulated mortality rate of CCA has increased by 39% [2]. In Asia and South America, the incidence rate of CCA is 96 cases per 100,000, which is even higher than that of US [3]. The significantly increased incidence, high mortality rate and poor prognosis of CCA have attracted increasing research interest in recent years [1, 4]. Unfortunately, due to the early occurrence regional nodal and distant metastases, only 10% of CCA patients are surgically resectable and curable, which leads to the poor prognosis with a median survival of less than one year [1, 5].

The profound genetic heterogeneity, highly desmoplastic nature and rich tumor microenvironment of CCA are thought to account for its early and extensive onset of metastasis [1, 6, 7]. For example, Yoshikawa et al. reported that the epidermal growth factor receptor overexpression was associated with lymph node metastasis, lymphatic vessel invasion and perineural invasion in extrahepatic CCA, and vascular endothelial growth factor overexpression with intrahepatic metastasis in intrahepatic CCA [8]. In addition, several studies have indicated that epigenetic modifications of genes involved in progression turned out to be another possible mechanism underlying CCA metastasis [9]. For instance, E-cadherin promoter methylation was found to be associated with increased migration and invasion in CCA [9–14]. Nonetheless, the mechanisms related to CCA metastases remain incompletely understood.

CCA is located near the omentum and mesentery, therefore surrounded by a rich tumor microenvironment composed of heterogeneous stromal cells including adipocytes [15]. Adipocytes are one of the key components of the supportive microenvironment for nearby tumor cells, which functions to serve as an energy source by releasing free fatty acids (FAs), as well as to derive a wide variety of adipokines and signaling factors involved in tumor formation and progression [15–19]. Recently, adipocytes have been regarded to be involved in regulating matrix remodeling, invasion, and epithelial-mesenchymal transition (EMT), which is a pivotal mechanism for tumor metastatic dissemination [7, 20, 21], in several human cancers [22, 23]. For example, Nieman et al. demonstrated that adipocyte-derived FAs and soluble factors promoted the homing, migration and invasion of ovarian cancer cells in 2011 [18]. Also, enhanced lipolysis and increased β-oxidation were respectively observed in adipocytes and co-cultured ovarian cancer cells. Afterwards, growing evidences showed that adipocytes promoted proliferation and progression of several malignancies such as colon, breast, and renal cancers etc. [24–28].

Although the association between obesity and CCA has been reported in a meta-analysis in 2014 [29], the association between adipocytes and CCA progression remains unknown, not to mention the underlying mechanisms. To identify the association between adipocytes and CCA metastasis, the present study demonstrated, for the first instance, the effects of adipocytes on CCA metastasis and the related mechanism, which may reveal a potential therapeutic target against CCA progression.

## Methods

### Adipose tissues and preparation of extracts

Human adipose tissues from the breast cancer patients (> 2 cm away from tumors) and the omentum majus adipose from the nontumorous patients were provided by the First Affiliated Hospital of Soochow University less than 1 h after surgery. This study was approved by the Institutional Review Board of Soochow University. Under aseptic conditions, the adipose tissues were washed with icy-cold PBS containing 50 μg/ml gentamicin, cut into small pieces with a diameter of 2 mm, and then centrifuged briefly to remove red blood cells and debris. Adipose tissues then were incubated in DMEM culture medium (80 mg adipose/mL DMEM medium) for 24 h at 37 °C under 5% CO_2_. Adipose tissue extracts were obtained by removing lipochondrions via 0.45 μM filter filtration (EMD Millipore, Billerica, MA, USA). After incubation with the extracts for 24 h, the cancer cells were harvested for subsequent experiments.

### Pre-adipocyte differentiation induction assay

A differentiation assay was performed as previously described [30]. Briefly, 3 T3-L1 cells were first incubated in differentiation media I consisting of DMEM, 10% fetal bovine serum (FBS), 0.25 μg/mL insulin, 1 μM dexamethasone, and 0.5 mM 3-isobutyl-1-methylxanthine (Sigma-Aldrich, St. Louis, MO, USA). Two days later, the cells were cultured in complete culture medium and 0.25 μg/mL insulin (differentiation media II) until lipid droplets formed. Oil Red O staining (Abcam, Cambridge, MA, USA) was employed to determine differentiation status as previously described [30]. The supernatant was collected prior to lipid droplet staining through centrifugation; the supernatant was used in subsequent experiments.

### Cell culture, drug treatment and adenovirus infection

The human CCA cell lines RBE and Hccc-9810 were maintained in RPMI 1640 containing 10% FBS, L-glutamine (2 mM), non-essential amino acids (2 mM), penicillin (100 U/mL), and streptomycin (100 U/mL) (Gibco, Grand Island, NY, USA) at 37 °C under 5% CO_2_ atmosphere. BMS309403 (Sigma-Aldrich, St. Louis, MO, USA) was dissolved in DMSO (Sigma-Aldrich, St. Louis, MO, USA) at 20 mM to prepare a stock solution. In the subsequent studies, 20 μM BMS309403 was used as working solution.

Full-length cDNA of an adenovirus-carrying human FABP4 was constructed for in vivo expression of FABP4 (ViGene, Shandong, China). Sub-confluent cells were mock-infected or infected with adenovirus in complete medium for 12 h at 37 °C followed by incubation in fresh complete medium for additional 24 h to 48 h for subsequent experiments. The infection efficiency was confirmed by detection of FABP4 mRNA by quantitative real-time PCR (qRT-PCR).

### Wound healing migration assay

3 × 10^5^/mL exponentially grown CCA cells were split and seeded into 6-well tissue culture plates and allowed to form a confluent monolayer. After a corresponding treatment, the monolayer was scratched with the tips of 200 μL sterile pipettes, washed with PBS to remove floating and detached cells, and cultured with fresh medium supplemented with 2% FBS. To assess cell migration, we designated five randomly chosen points for each treatment group for photographing at 0, 12, 24, and 48 h by using a microscope (40×, Olympus, Tokyo, Japan) equipped with a digital camera (Canon, Tokyo, Japan); the images were analyzed using Image J software (National Institutes of Health, Bethesda, MD, USA). Cell migration was presented as means ± sd of wound width, and the values were compared with those at the starting time point in the control group.

### Transwell invasion assay

The invasion assay was performed using 24-well Matrigel Invasion Chambers (pore size, 8 μm; Corning, Tewksbury, MA, USA). Inserts were pre-coated with 50 μL of Matrigel (1:8 dilution; BD Biosciences, San Jose, CA, USA) and then polymerized at 37 °C for 4 h before the experiment. 5 × 10^4^/100 μL CCA cells were prepared as a single cell suspension in serum-free medium containing 0.2% bovine serum albumin (BSA) and seeded onto the upper chambers. The lower chamber was filled with complete culture medium supplemented with/without adipose tissue extracts or filled with culture supernatant from differentiated 3 T3-L1 cells. After incubation for 24 h, the cells on the surface of the upper chambers were scraped off. The invading cells were fixed with 4% paraformaldehyde, stained with Giemsa staining solution at room temperature for at least 4 h, and photographed under a microscope.

### Free FA assay

Cells were plated into a 24-well plate and treated accordingly. The medium was collected and centrifuged at 10,000 *g* for 5 min at 4 °C prior to determination. Non-esterified FA (NEFA) in the culture media was analyzed using colorimetric assays according to the manufacturer’s instructions (Labassay NEFA Kit, Wako, Osaka, Japan).

### Immunofluorescence staining

Cells were grown on 35 mm glass bottom culture dishes (Nest Scientific, NJ, USA). After the corresponding treatments, the cells were washed with PBS and fixed with 4% paraformaldehyde, stained with 20 μg/mL Bodipy 493/503 (ThermoFisher Scientific, Grand Island, NY, USA) for 1 h at room temperature, and washed twice with PBS. The nuclei were visualized using Hoechst 33342 (0.5 μg/mL), and the stained cells were observed under a confocal scanning laser microscope (Olympus, Tokyo, Japan). The relative fluorescence intensity was analyzed using Image J software.

### Western blot assay

Cells were harvested and lysed in 50 uL of lysis buffer containing protease inhibitor cocktail (Roche Life Science, Indianapolis, IN, USA) for 30 min on ice. Total protein (50 μg) from each lysate was fractionated by 10% SDS-PAGE and transferred onto polyvinylidene fluoride microporous membranes. After blocking with 5% nonfat milk in PBS-Tween-20 for 2 h at room temperature, the membranes were incubated with primary antibody overnight at 4 °C and then incubated with corresponding secondary antibodies. GAPDH was used as loading control.

The primary antibodies used were rabbit anti-FABP4 (EPR3579, Abcam, Cambridge, MA, USA, 1:1000), rabbit anti-claudin 1 (Abcam, Cambridge, MA, USA, 1:1000), rabbit anti-occludin (EPR8208, Abcam, Cambridge, MA, USA, 1:50,000), rabbit anti-E-cadherin (EP700Y, Abcam, Cambridge, MA, USA, 1:10,000), rabbit anti-SNAIL (Abcam, Cambridge, MA, USA, 1:1000), rabbit anti-Smad3 (EP568Y, Abcam, Cambridge, MA, USA, 1:1000), rabbit anti-β-catenin (Cell Signaling Technology, Danvers, MA, USA, 1:1000), rabbit anti-MMP2 (Cell Signaling Technology, Danvers, MA, USA,1:1000), rabbit anti-MMP9 (Cell Signaling Technology, Danvers, MA, USA,1:1000), and anti-β-actin (Beyotime Biotechnology, Haimen, China). The secondary antibodies used were goat anti-mouse and anti-rabbit horseradish peroxidase-conjugated antibodies (1:1000, Beyotime Biotechnology, Haimen, China). Protein expression was quantified using Image J software.

### qRT-PCR assay

Total RNA from each group of RBE cells was extracted using TRIzol (Invitrogen, Grand Island, NY, USA) according to the standard TRIZOL method. First-strand cDNA was synthesized from 1 μg of RNA per sample by using Transcriptor First Strand cDNA Synthesis Kit (Roche Life Science, Indianapolis, IN, USA). Real-time PCR was performed on an Applied Biosystems ViiA 7 RT-PCR by using SYBR Green (Thermofisher Scientific, Grand Island, NY, USA). Table 1 shows the primers for FABP1–7 and the internal control GAPDH. Gene expression was calculated using the 2^-△△CT^ method.

**Table 1:** Primers for qRT-PCR of FABP1 to 7

Gene	Forward primer sequence (5′-3′)	Reverse primer sequence (5′-3′)
FABP1	AGTGGTTCAGTTGGAAGGTGA	GCAGACTTGTTTAAATTCTCTTGC
FABP2	ATTTCCATTCATGCCAAAG	TCCACTACATTCCAGCCTGA
FABP3	ACACTTGTGCGGGAGCTAAT	CATGGGAACTGGAACTGGAT
FABP4	ACCTTAGATGGGGGTGTCCT	ACGCATTCCACCACCAGTTT
FABP5	ACAGATGGTGCATTGGTTCA	CCTGTCCAAAGTGATGATGG
FABP6	CTCAGAGATCGTGGGTGACA	CGAGCAGCGTCTGTCCTTAT
FABP7	ACAGAAATGGGATGGCAAAG	ATTTTCCACCTCCACACCAA
GAPDH	CAGGAGGCATTGCTGATGAT	GAAGGCTGGGGCTCATTT

### In vivo analysis of CCA metastasis in nude mice

Five-week-old female outbred nude mice were purchased from Shanghai SLAC Laboratory Animal Co., Ltd. (Shanghai, China) and housed in a pathogen-free facility with constant temperature and humidity control. CCA metastasis mouse models were constructed by injecting 1 × 10^6^/100 μL RBE cells through the tail vein. Briefly, the mice were randomly divided into five groups, which were separately injected with (1) parental RBE cells, (2) RBE cells co-cultured with human omentum adipose for 24 h, (3) RBE co-cultivated with human adipose and FABP4 inhibitor BMS309403 for 24 h, and (4 and 5) RBE cells infected with control adenovirus and FABP4 adenovirus for 24 h, respectively. For short-term imaging assay, all cells were infected with green fluorescent protein (GFP)-tagged adenovirus for additional 24 h, collected and washed twice with cold PBS, and then re-suspended in cold PBS. 24 h after injection, colonization of RBE cells in organs of nude mice was visualized using an in vivo imaging system (Kodak, Effingham, IL, USA). All photos were taken under the same condition. The relative fluorescence intensity of the tissues was calculated using Image J analysis software. For long-term observation of metastasis, all mice were euthanized 1 month after injection through the tail vein, and their livers and lungs were extirpated and used in pathological examination as described below. The design and implementation of this study were approved by the Ethics Committee of Soochow University.

### Histopathological and immunohistochemistry assay

All of the animals were euthanized 1 month after injection through the tail vein. The liver, heart, kidney, and lung of each mouse were excised. Tissues were fixed in 10% neutral-buffered formalin and embedded in paraffin for H & E staining and immunohistochemistry as previously described [31] to determine the metastasis of RBE cells in vivo.

For immunohistochemistry, 4 μm paraffin sections were deparaffinized and heat-treated with citrate buffer (pH 6.0) for 3 min according to an antigen retrieval protocol. Non-specific binding sites were subsequently blocked with 5% BSA for 30 min. The sections were incubated overnight with primary antibodies (1:200 dilution) at 4 °C. Subsequently, the sections were incubated with secondary antibody dilution (Zhongshan Golden Bridge Biotechnology, Beijing, China) at 37 °C for 1 h followed by diaminobenzidine (DAB) substrate detection, washing, hematoxylin staining, dehydration and mounting. Images of the tissue sections were captured using an Olympus optical microscope (Tokyo, Japan).

For Oil Red O staining, 10 μm frozen sections made from fresh frozen tissues were air-dried at room temperature, incubated in fresh Oil Red O (Abcam, Cambridge, MA, USA) for 10 min, and rinsed in water. Images of the slides were viewed and captured under an Olympus optical microscope (Tokyo, Japan).

### Statistics

All experiments were performed in triplicate and data were expressed as mean ± sd. SPSS Statistics (Version 19.0, IBM, Armonk, NY, USA) was used for student’s t-test or one-way ANOVA to evaluate differences. *P* < 0.05 indicates significant difference.

## Results

### Adipose tissue extracts enhance CCA invasion and migration in vitro

**Fig. 1:**
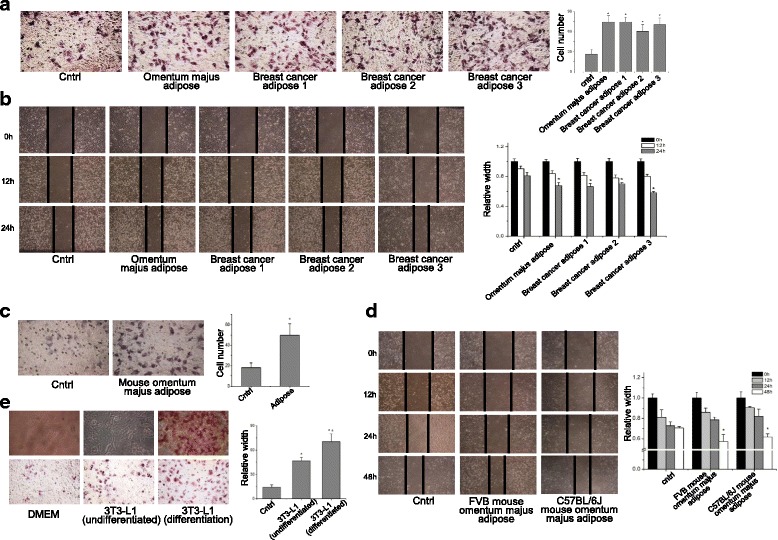
Human and mouse adipose tissue extracts promote the invasion and migration of CCA cells in vitro. Human cholangiocarcinoma (CCA) cell line RBE was utilized for in vitro invasion and migration analysis. To determine invasion, RBE cells were seeded onto the top chamber coated with matrigel, while the complete growth medium or growth medium containing adipose tissues extracts (80 mg/mL) from one human omentum majus and 4 breast cancer associated adipose tissues (**a**), FVB and C57/BL6J mice omentum majus adipose (**c**), and culture supernatant for differentiated 3 T3-L1 cells (**e**) were filled in the lower chambers. After additional incubation for 24 h, the invading RBE cells were fixed, visualized, and quantified. To determine migration, confluent monolayer of RBE cells were scratched and incubated with complete growth medium, human adipose extracts (**b**), and mice adipose extracts (**d**) for another 12 h, 24 h, or 48 h. The wound closure over time was monitored and presented as the ratio to culture medium incubated control group. All the samples were prepared in triplicate, and all experiments were repeated at least three times. *P < 0.05; **P < 0.01

Tumor microenvironment, which consists of tumor-associated extracellular matrix and stromal cells, is important for tumor growth and progression [32]. To determine the effects of adipose tissues, a major component of tumor microenvironment, on CCA invasion and migration, we incubated the human CCA cell line RBE with extracts of adipose tissues obtained from different species or origins. Figure 1a showed that the invasion of RBE cells, cultured in the presence of the four human adipose tissue extracts, was significantly increased compared with those of the control group (*P* < 0.05), indicating that the acquired invasiveness of CCA cells was due to cultivation with adipose tissue extracts. Moreover, enhanced migration of RBE cells cultured with adipose tissue extracts was also detected by wound healing assay (Fig. 1b), in which the enhanced migration was indicated by obvious shrinking of “wound” width 24 h following scratching compared with that in the control group (P < 0.05). Enhanced invasion and migration were also observed in RBE cells grown in extracts of adipose tissue from C57/BL6J omentum (Fig. 1c and d).

Adipose tissues consist mainly of mature adipocytes, which play potential roles in ovarian cancer and breast cancer progression [18, 32]. In the present study, mouse pre-adipocyte 3 T3-L1, which can differentiate into mature adipocytes [30], was utilized to determine the effects of adipocytes on RBE metastasis in vitro. As shown in Fig. 1e, pre-adipocyte 3 T3-L1 could be induced to differentiate into mature adipocytes, characterized by the presence of typical lipid droplets in the cytoplasm [30]. Compared with RBE cells grown in complete growth medium, RBE cells co-cultured with undifferentiated 3 T3-L1 exhibited a stronger invasive tendency (*P* < 0.05). Moreover, co-cultivation with fully differentiated 3 T3-L1 cells significantly increased the number of trans-membrane cells, in contrast to pre-adipocyte-co-cultured RBE cells (*P* < 0.05). Overall, these results above demonstrated for the first time that crosstalk of CCA cells with adipose tissues and mature adipocytes was essential for CCA metastasis in vitro.

### Adipose tissue extracts promote EMT of CCA cells

**Fig. 2:**
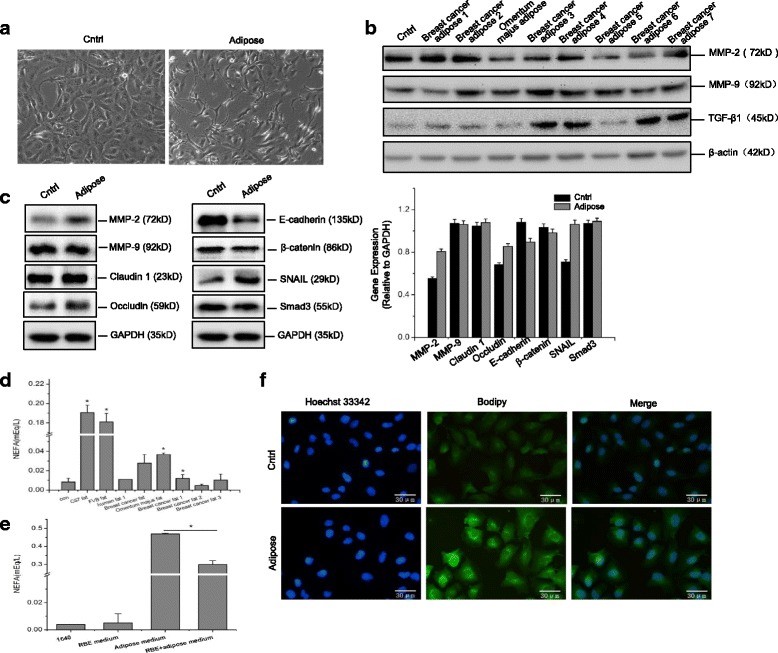
Adipose tissue extracts enable the EMT of CCA cells. Human cholangiocarcinoma (CCA) cell line RBE was incubated with complete growth medium or medium containing human adipose tissue extracts for 24 h. The cell morphology was observed under the invert microscopy (40×) (**a**). Protein expression of MMP2, MMP9, TGF-β1, claudin 1, occludin, E-cadherin, β-catenin, SNAIL, and Smad3 were analyzed by immunoblot analysis in response to adipose extracts incubation, and the band densitometry analysis was carried out relative to the loading control β-actin or GAPDH (**b**, **c**). To determine fatty acid metabolism, the culture medium with different adipose components prior to or after incubation with RBE cells for 24 h were collected for extracellular glycerol and nonestesterified fatty acid (NEFA) detection using colorimetric assays, with the data presented as the absorbance at 490 nm(**d**, **e**). The intracellular lipids in adipose cultured RBE cells were visualized by the fluorescence dye Bodipy (20 μg/mL) using confocal microscopy analysis, using Hochest33342 (0.5 μg/mL) for nuclear staining (**f**). All the samples were prepared in triplicate, and all experiments were repeated at least three times. *P < 0.05

EMT is a pivotal mechanism of tumor metastasis in many epithelial malignancies [7, 20]. In the present study, besides enhanced RBE metastasis in vitro, altered morphologies, from cobblestone-like to typical spindle-like, were found in RBE cells cultivated in adipose tissue extracts (Fig. 2a). To determine whether adipose tissues influence CCA metastasis by regulating EMT signal pathways, we detected related protein markers in RBE cells cultured in the presence or absence of adipose tissue extracts via Western blot assay. Figure 2b and c showed the enhanced EMT phenotype markers in RBE cells cultivated in adipose tissue extracts, including upregulation of matrix metalloproteinase (MMP)-2, MMP-9 and SNAIL, as well as downregulation of E-cadherin and β-catenin. These findings are consistent with the results of Cadamuro et al., who reported that CCA bile ducts expressed several EMT phenotype markers [33].

Furthermore, we detected significantly enhanced expression of transforming growth factor β1 (TGF-β1) in RBE cells cultured with extracts of adipose tissues from different origins (Fig. 2b, Additional file 1 Figure S5A). TGF-β1 is known to promote EMT by inducing SNAIL, and is very important in reprogramming of cellular energy in cancers to satisfy the increased energy demand during EMT to sustain enhanced mobility [34]. Triglycerides (one molecule glycerol plus 3 molecules FA), being the major cellular energy source, are mainly stored in mature adipocytes [35–37]. To investigate whether adipose tissue extracts promoted EMT phenotype of CCA cells by affecting cellular energy homeostasis, we examined extracellular glycerol and nonestesterified fatty acid (NEFA) in RBE cells before and after cultivation with different adipose tissue extracts. As shown in Fig. 2d, e and Additional file 1 Figure S1, significantly increased amounts of glycerol and NEFA were detected in the adipose tissue extract-containing culture medium (*P* < 0.05), compared with the regular culture medium. In addition, we found that it is NEFA, rather than glycerol, that was significantly reduced after cultivating RBE cells in culture medium containing adipose tissue extracts (Fig. 2d and e, *P* < 0.05). Moreover, enhanced lipid accumulation was observed in RBE cells cultivated in adipose tissue extracts (Fig. 2f), consistent with findings suggesting that FA plays a key role in certain pathological processes by promoting cancer cell lipogenesis [38, 39]. Overall, the results above indicated that adipose tissue facilitates EMT of CCA cells in vitro, likely by providing NEFAs as energy source.

### Adipose tissue extracts enhance CCA metastasis in nude mice

**Fig. 3:**
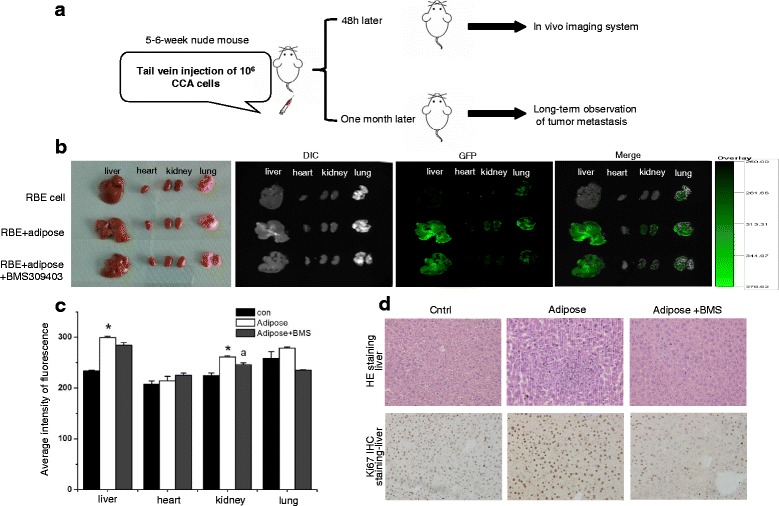
Adipose tissue extracts facilitate CCA metastasis in nude mice. RBE cells (1 × 10^6^) were separately incubated in growth medium, adipose extracts medium, and adipose extracts plus fatty acid uptake inhibitor BMS309403, and then injected through the tail vein of Balb/C nude mice to construct the CCA metastasis mice model (**a**). 24 h after inoculation, the in vivo migration and invasion were observed using the living animal imaging system (**b**), and were quantified by fluorescence intensity (**c**). One month after injection, mice were euthanized. Histopathological analysis of livers and lungs were performed on formaldehyde fixed-paraffin embedded sections via H&E staining (**d**). *n* = 5 per group, *P < 0.05; ^a^ *P* < 0.01

The influence of adipose tissues on CCA metastasis was further confirmed in vivo in CCA metastasis Balb/C nude mouse model (Fig. 3a). First, 48 h after inoculation, distribution and accumulation of RBE cells were visualized using an in vivo imaging system. As shown in Fig. 3b and c, significantly increased fluorescence signals were detected in the liver and kidney of mice injected with RBE cells cultivated in adipose tissue extracts, compared with mice injected with RBE cells under normal culture conditions (*P* < 0.05). However, the chemical compound BMS309403, which could specifically inhibit FA uptake in vitro [40], compromised the adipose-induced CCA metastasis in vivo, especially in the kidney (*P* < 0.05).

One month after inoculation, 40% (2/5) of the mice injected with RBE cells cultivated in adipose tissue extracts developed liver metastasis, in which the hepatocytes manifested typical pathologic characters, such as irregular shape, variable sizes, hyperchromatic and pleomorphic nuclei, nuclear disintegration and increased proliferation (Fig. 3d). Moreover, no distance metastasis and obviously suppressed cancer proliferation were observed in BMS309403 plus adipose tissue-cultivated RBE injected mice, compared with adipose tissue extract-cultivated RBE group (Fig. 3d). The results above are consistent with the in vitro data showing that adipose tissue can promote CCA metastasis.

### FABP4 is involved in CCA metastasis

**Fig. 4:**
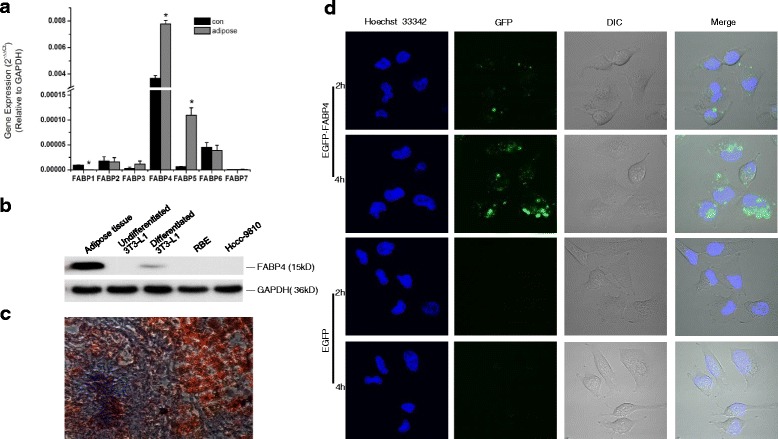
Fatty acid binding protein 4 (FABP4) is involved in the metastasis of CCA. Human CCA cell RBE was treated with or without human adipose tissue extracts for 24 h. The mRNA levels of FABP 1 to 7 were determined using qRT-PCR with β-actin as control gene (**a**). To detect the protein expression of FABP4, 50 μg of human adipose tissues, undifferentiated or fully differentiated pre-adipocyte 3 T3-L1, as well as CCA cell RBE and Hccc-9810 were analyzed by Western blot assay, using GAPDH as loading control (**b**). To measure CCA tissues contained lipids, patient CCA tissues (*n* = 3) were freshly made into 5 μm frozen sections, stained using OilRed solution, and observed under the microscopy (40×) (**c**). To monitor the uptake of exogenous FABP4 protein by CCA cells, exponential monolayer RBE was incubated with synthesized EGFP-tagged FABP4 for 2 h or 4 h, using EGFP as a control. The intracellular distribution of FABP4 was visualized using the living cell imaging system (**d**). All the samples were prepared in triplicate, and all experiments were repeated at least three times. *P < 0.05

Based on the data above, we speculated that FAs released by adipocytes might at least be involved in adipose-induced CCA metastasis. However, free FAs are relatively insoluble and potentially toxic, and they require non-catalytic binding proteins to perform their biological functions [41]. FABPs are a family of intracellular proteins that exhibit high affinity for noncovalent binding to long-chain FAs [40]. In the present study, we detected the expression of FABP1–7 in RBE cultured with/without adipose tissue extracts by qRT-PCR assay. The result revealed that adipose coculture significantly enhanced the expression of FABP5 and FABP4, while decreased FABP1 expression in RBE cells, as compared with cells cultured in normal complete medium (Fig. 4a). We noticed that, of these three FABPs, FABP4 was the most enriched one in RBE cells under normal culture condition. Because FABP4 has been regarded as an essential adipokine mostly derived from adipocytes [41], it was chosen for further study in the present work.

As expected, we demonstrated that FABP4 highly expressed in human omentum adipose and mature adipocytes, but not in human CCA tissues or cell lines (Fig. 4b and Additional file 1 Figure S2). Coincidently, human CCA tissues were shown to containing more adipocytes than non-cancerous tissues (Fig. 4c). Taken together the finding that synthesized EGFP-tagged FABP4 protein could be taken up by CCA cells (Fig. 4d), we speculated that the adipocyte-derived FABP4 might play a role in mediating adipocyte-induced CCA metastasis.

### FABP4 inhibition disrupts adipocyte-induced CCA metastasis and EMT

**Fig. 5:**
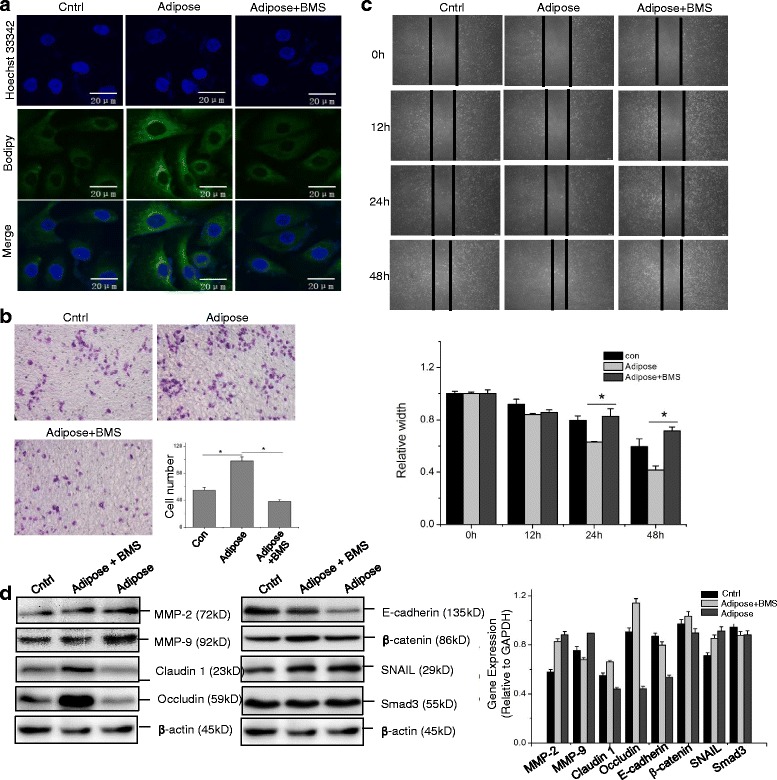
Inhibition of FABP4 suppresses the metastasis of RBE cells. Parental RBE cell and RBE treated with adipose extracts or adipose extracts plus 20 μM/L BMS309403 for 24 h were utilized for the following analysis. The immunofluorescence staining of intracellular lipids was determined using fluorescence dye Bodipy under the confocal microscopy (**a**). The invasiveness and migration of RBE cells with different culture condition was determined via transwell invasion assay (**b**) and wound healing assay (**c**) as described above. The EMT related protein expression was detected via Western blot assay (**d**). All the samples were prepared in triplicate, and all experiments were repeated at least three times. *P < 0.05

**Fig. 6:**
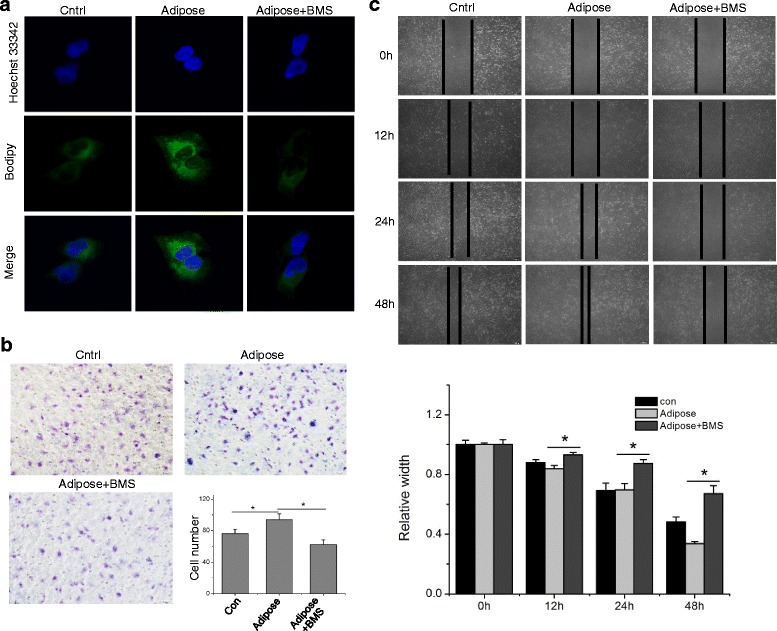
FABP4 inhibitor suppresses the invasion and migration of Hccc-9810 cells in vitro. Hccc-9810 cells were treated with adipose extracts or adipose extracts plus 20 μM/L BMS309403 for 24 h, with parental cell as untreated control. The intracellular lipids were determined by Bodipy immunofluorescence staining confocal microscopy (**a**). The transwell invasion assay (**b**) and wound healing assay (**c**) were applied to detect the invasion and migration of Hccc-9810 in vitro. All the samples were prepared in triplicate, and all experiments were repeated at least three times. *P < 0.05

To test our above hypothesis, we used the specific FABP4 inhibitor BMS309403 in the following study (Additional file 1 Figure S3A). BMS309403 effectively inhibited the lipid accumulation in both adipose-cocultured RBE and Hccc-9810 cells (Additional file 1 Figure S6, Fig. 5a, 6a), and significantly suppressed the adipose-induced CCA invasion and migration in vitro. For example, the number of invading RBE cells cultivated in adipose tissue extracts was nearly 2-fold of that in control. This trend can be completely reversed by adding FABP4 inhibitor to the adipose tissue extract-containing medium (Fig. 5b). Moreover, the promoted migration of RBE cells cultured with adipose tissue extracts could be suppressed by BMS309403 treatment (Fig. 5c). A similar tendency was observed in Hccc-9810 cells as well (Fig. 6).

Western blot assay results showed that MMP-2, MMP-9 and the EMT-associated SNAIL transcription factor were upregulated, whereas the tight junction proteins claudin1 and occludin were downregulated. Moreover, E-cadherin and β-catenin were suppressed in RBE cells cultivated in adipose tissue extracts compared with that in parental RBE cells (Fig. 5d). However, the BMS309403 treatment completely reversed the expression patterns of the above mentioned EMT marker proteins.

### FABP4 overexpression enhances EMT and adipocyte-induced CCA metastasis

**Fig. 7:**
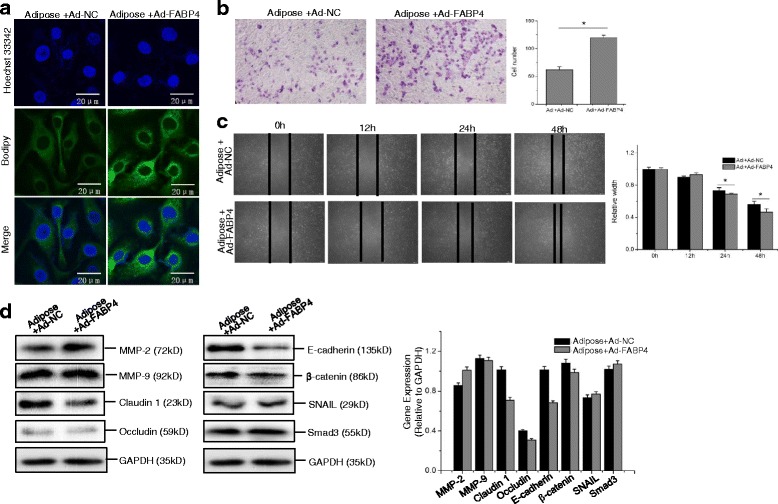
FABP4 overexpression enhances the in vitro metastasis of RBE cells. RBE cells were infected with FABP4 recombinant adenovirus (1:100) and control adenovirus for 24 h, and then were incubated in adipose extracts for additional 24 h. To measure the intracellular lipids, Bodipy immunofluorescence staining was performed as described above (**a**). The in vitro invasion (**b**) and migration (**c**) of Hccc-9810 were analyzed and quantified as described above. Western blot assay was performed to detect the expression levels of EMT related protein (**d**). All the samples were prepared in triplicate, and all experiments were repeated at least three times. *P < 0.05

**Fig. 8:**
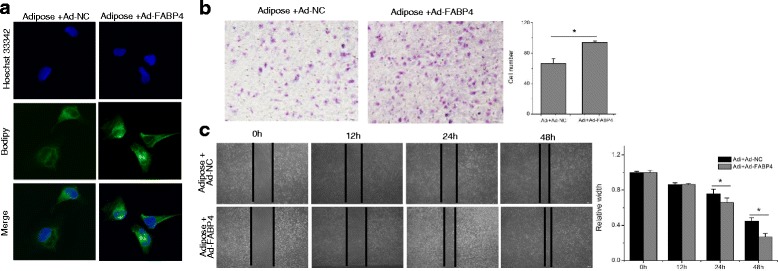
Overexpression of FABP4 promotes the in vitro metastasis of Hccc-9810 cells. Hccc-9810 cells were treated with FABP4 recombinant adenovirus (1:100) and control adenovirus infection and adipose extracts cultivation. Bodipy immunofluorescence staining was performed to measure the intracellular lipids (**a**). The transwell invasion assay (**b**) and wound healing assay (**c**) were conducted to analyze the in vitro metastasis of Hccc-9810. All the samples were prepared in triplicate, and all experiments were repeated at least three times. *P < 0.05

To further confirm the role of FABP4 in CCA metastasis, FABP4 recombinant adenovirus was used to demonstrate the involvement of FABP4 in adipocyte-modulated CCA progression (Additional file 1: Figure S3). FABP4 overexpression enhanced the intracellular lipid accumulation, increased the number of invading cells and the rate of wound healing both in RBE and Hccc-9810 cells (Figs. 7 and 8), and promoted the early metastasis of RBE cells in vivo (Additional file 1 Figure S4). Expressions of EMT-related protein markers were examined in FABP4-overexpressing RBE cells via Western blot assay. As expected, FABP4 overexpression further boosted the expression levels of MMP-2 and SNAIL, whereas it inhibited the expressions of tight junction protein and E-cadherin/β-catenin proteins, compared with that in adenovirus vector control group (Fig. 7d). However, despite these differences, no obvious variations in Smad3 was detected in all the treatments (Fig. 5d and 7d). These results collectively indicated that FABP4 was involved in adipose tissue-mediated CCA metastasis.

## Discussion

As the major components of adipose, adipocytes play a key role not only in controlling metabolism homeostatic, but also in mediating several pathological processes by releasing lipids and mitogenic and proinflammatory factors [42]. Lately, cancer-associated adipocytes have been shown to regulate tumor progression in hepatocarcinoma [43], ovarian, and breast cancers [18, 25, 32, 44]. Several reports have indicated that adipose tissues from different anatomic location may produce different cytokines and growth factors, therefore they behave not exactly the same way [45, 46]. Especially in couple of breast cancer studies, estrogen receptor positive (ER^+^) adipose tissues obtained from breast cancers were found to behave differently in ER^+^ breast cancer cell line MCF7, as compared to adipose from normal breast [26, 47].

However, we demonstrated in the present study that different adipose tissue and mature adipocytes significantly enhanced the invasion and migration of CCA both in vitro and in vivo, regardless of the species and anatomical locations from which the adipose tissues were isolated. Considering that the biological effects of adipocytes on CCA cells are remain unknown, we speculated that certain potential mechanisms complementary to the known ones might at least be involved in adipocyte induced CCA metastasis.

The involvement of adipocytes in tumor progression was thought to be mediated via regulating factors involved in matrix remodeling, invasion and survival of cancer cells, as well as inducing EMT [15]. EMT is a dynamic process, through which epithelial cells structurally and functionally gain the mesenchymal characteristics, and has lately been regarded as a pivotal mechanism for tumor metastatic dissemination [7, 20, 21]. During this process, EMT-inducing transcription factors (SNAIL, ZEB and TWIST etc.) regulate the expressions of E-cadherin and β-catenin, as well as genes associated with mesenchymal phenotypes including N-cadherin, vimentin, fibronectin, α-smooth muscle actin (α-SMA) and MMPs, which leads to the formation of several migratory structures and the degradation of the extracellular matrix [21, 48, 49]. Together with the compromised tight junction, a repressed epithelial phenotype is finally replaced by an activated mesenchymal phenotype, which enables the tumor cells with invasive properties to spread toward other tissues/organs [21, 49].

In CCA, several reports have shown that cancer-associated fibroblasts and tumor-associated macrophages play key roles in CCA progression [21]. However, the present study is the first one to report that co-culture with adipocytes could induce the representative mesenchymal properties in CCA cells, which resulted in alterations in cell polarity, cell shape, loss of cell-to-cell adhesion regulated by E-cadherin, and expressions of mesenchymal biomarkers, indicating the acquired EMT induced by CCA associated adipocytes.

Typically, cancer cells are characterized with accelerated proliferation and aggressive phenotypes, therefore they demand enhanced energy metabolism by increasing de novo lipogenesis [50, 51]. When de novo lipogenesis is inhibited in some cases [52, 53], the adipocytes from the tumor microenvironment are known to serve as a major source to provide exogenous lipids for survival of tumor cells [54]. For example, adipocytes-derived FAs are known to serve as economical and important lipid sources to meet the higher energy requirement for enhanced mobility of ovarian cancer cells [55]. In the present study, the increased uptake of FAs and enhanced intracellular lipid accumulation were observed in adipose extract-co-cultured CCA cells, which present typical EMT phenotype. These results indicated that adipocyte-derived FAs might be one of the regulators potentially involved in adipocyte-induced EMT in CCA, which are consistent with previous study that free FAs exacerbate the EMT phenotypes in hepatocellular carcinoma cells [56].

However, relatively insoluble free FAs require non-catalytic binding proteins to perform the biological functions mentioned above [41]. In the present study, we found certain members of fatty acid binding protein family might be involved in this process. For example, significantly increased adipocyte FABP (FABP4) and epithelial FABP (FABP5), as well as suppressed liver FABP (FABP1) in adipose cocultured CCA cells. Firstly identified in liver tissues, FABP1 was thought to be vital for hepatic β-oxidation of unesterified fatty acid, and FABP1 downregulation was reported to be associated with hepatic stellate cells activation, proliferation, and secretion of collagen and extracellular matrix protein, which would finally lead to fibrogenesis [57]. FABP5 expression was proved to be detectable in endothelial cells, lung epithelium, macrophages, adipocytes, and breast cancer cells [57]. FABP5 Upregulation has been related to promoted cancer cell proliferation and metastasis, therefore FABP5 also known as oncogenic FABP [57]. These evidences indicated that FABP1 and FABP5 might be possible regulatory mechanisms in adipocyte-induced CCA metastasis.

**Fig. 9:**
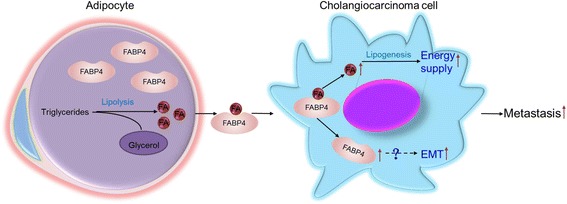
Adipocytes interact with cholangiocarcinoma cells to induce metastasis in cancer cells. FABP4 mediates the increased adipocyte-derived FAs absorption in CCA cells, providing energy for more aggressive phenotypes in CCA. Furthermore, adipocytes-derived FABP4 itself plays a regulatory role in CCA metastasis

However, FABP4 was selected for further analysis in the present work. Because FABP4 mainly presents in adipose tissues or mature adipocytes, possesses the highest affinity among all FABPs for both saturated and unsaturated FAs, and its functions in intracellular metabolism homeostasis and immunometabolic diseases are the best characterized [41]. Besides, FABP4 has also been shown to be an adipose-derived cytokine that could be released into circulation [41, 58], and involved in cancer cell growth and metastasis in multiple malignancies [18, 41, 59]. For example, Nieman et al. observed highly expressed FABP4 in omental metastases but not in primary ovarian cancers [18]; Uehara et al. also reported that FABP4 could promote human prostate cancer cell progression by binding to FAs [59]. In the present study, both in vitro and in vivo data clearly showed that the adipocyte-derived FABP4 mediates the adipose-induced intracellular lipid accumulation, invasion, migration, and EMT in CCA cells, at least by acting as FA transporter between adipocytes and tumor cells (Fig. 9).

## Conclusions

In summary, our current study, for the first time, demonstrates the roles of FABP4 in adipocyte-CCA interactions, as well as in the energy extraction of CCA cells from cancer related adipocytes. FABP4 mediates the adipocyte-induced invasion, migration and EMT of CCA cells. Since the selective inhibitor of FABP4 blocks FABP4-mediated regulation on CCA metastasis, our findings provide a potential therapeutic target to interfere with CCA metastasis. However, CCA is a very complex and heterogeneous tumor with the prominent stromal components, therefore the EMT signaling could be activated through a large number of mechanisms, strong efforts and further experiments are eagerly needed to define the downstream signaling cascades of FABP4 involvement in adipocyte-induced EMT of CCA cells.

## Additional file

Additional file 1: Figure S1. Glycerol colorimetric assay. Human cholangiocarcinoma (CCA) cell line RBE was incubated in complete growth medium or adipose tissue extracts (80 mg/mL) supplemented medium for 24 h. The culture medium were collected prior to or after cell culture for extracellular glycerol detection using colorimetric assays, with the data presented as the absorbance at 490 nm (A,B). All the samples were prepared in triplicate, and all experiments were repeated at least three times. **P <* 0.05. **Figure S2.** The expression of FABP4 protein CCA cell RBE. Immunohistochemistry staining of FABP4 expression in cholangiocarcinoma tissue array**.** Human CCA tissue microarray was obtained from Outdo Biotech Co., Ltd. (Shanghai, China). The FABP4 antibody (EPR3579, Abcam, MA, USA, 1:200) was used for IHC staining. **Figure S3.** FABP4 expression in RBE and Hccc-9810 cells. Human CCA cell line RBE (A) and Hccc-9810 (B) were treated with BMS309403 (20 mg/mL or 40 mg/mL) for 24 h, as the same time these cell lines were infected with recombinant FABP4 adenovirus or control virus (1:10, 1:100), and mRNA expression of FABP4 was determined and are expressed relative to β-actin. All the samples were prepared in triplicate, and all experiments were repeated at least three times. **P* < 0.05. **Figure S4.** FABP4 overexpression affects CCA metastasis in vivo. 1 × 10^6^ RBE cells were infected with recombinant FABP4 adenovirus or control virus (1:10, 1:100) for 24 h, and incubated in adipose extracts medium for additional 24 h prior to tail vein injection of Balb/C nude mice. The in vivo migration and invasion of RBE cells were observed under the living animal imaging system 24 h after inoculation (A), and were quantified by fluorescence intensity (B). The mice were euthanized one month after injection. The livers and lungs were fixed in formaldehyde, embedded by paraffin, made into 4 μm sections, and stained using H&E assay (C). *n* = 5 per group, **P* < 0.05. **Figure S5.** Quantification of Western blot. The protein expression of TGF-β of RBE cells cocultured with different adipose tissues(A), and FABP4 in different samples (B) was quantified using Image J. The band densitometry analysis was carried out relative to the loading control β-actin. **P* < 0.05. **Figure S6.** Quantification of Bodipy staining. The intracellular lipids in adipose cocultured RBE cells (A), BMS309403 treated RBE cells (B), BMS309403 treated Hccc-9810 cells (C), FABP4 over-expressed RBE cells (D), and FABP4 over-expressed Hccc-9810 cells (E) were visualized by the fluorescence dye Bodipy (20 μg/mL), followed by fluorescence intensity analysis using Image J. All the samples were prepared in triplicate, and all experiments were repeated at least three times. **P* < 0.05. (DOCX 2322 kb)
