# Supplementary material for: Adipocytes promote cholangiocarcinoma metastasis through fatty acid binding protein 4
Source: J Exp Clin Cancer Res. 2017 Dec 13;36:183. doi: 10.1186/s13046-017-0641-y (PMC5729422; doi:10.1186/s13046-017-0641-y)
Supplement: Additional file 1: Figure S1. — Glycerol colorimetric assay. Human cholangiocarcinoma (CCA) cell line RBE was incubated in complete growth medium or adipose tissue extracts (80 mg/mL) supplemented medium for 24 h. The culture medium were collected prior to or after cell culture for extracellular glycerol detection using colorimetric assays, with the data presented as the absorbance at 490 nm (A,B). All the samples were prepared in triplicate, and all experiments were repeated at least three times. *P < 0.05. Figure S2. The expression of FABP4 protein CCA cell RBE. Immunohistochemistry staining of FABP4 expression in cholangiocarcinoma tissue array. Human CCA tissue microarray was obtained from Outdo Biotech Co., Ltd. (Shanghai, China). The FABP4 antibody (EPR3579, Abcam, MA, USA, 1:200) was used for IHC staining. Figure S3. FABP4 expression in RBE and Hccc-9810 cells. Human CCA cell line RBE (A) and Hccc-9810 (B) were treated with BMS309403 (20 mg/mL or 40 mg/mL) for 24 h, as the same time these cell lines were infected with recombinant FABP4 adenovirus or control virus (1:10, 1:100), and mRNA expression of FABP4 was determined and are expressed relative to β-actin. All the samples were prepared in triplicate, and all experiments were repeated at least three times. *P < 0.05. Figure S4. FABP4 overexpression affects CCA metastasis in vivo. 1 × 10^6^ RBE cells were infected with recombinant FABP4 adenovirus or control virus (1:10, 1:100) for 24 h, and incubated in adipose extracts medium for additional 24 h prior to tail vein injection of Balb/C nude mice. The in vivo migration and invasion of RBE cells were observed under the living animal imaging system 24 h after inoculation (A), and were quantified by fluorescence intensity (B). The mice were euthanized one month after injection. The livers and lungs were fixed in formaldehyde, embedded by paraffin, made into 4 μm sections, and stained using H&E assay (C). n = 5 per group, *P < 0.05. Figure S5. Quantification of Western blot. The protein expressio [file 13046_2017_641_MOESM1_ESM.docx]

***Supporting Information***

**
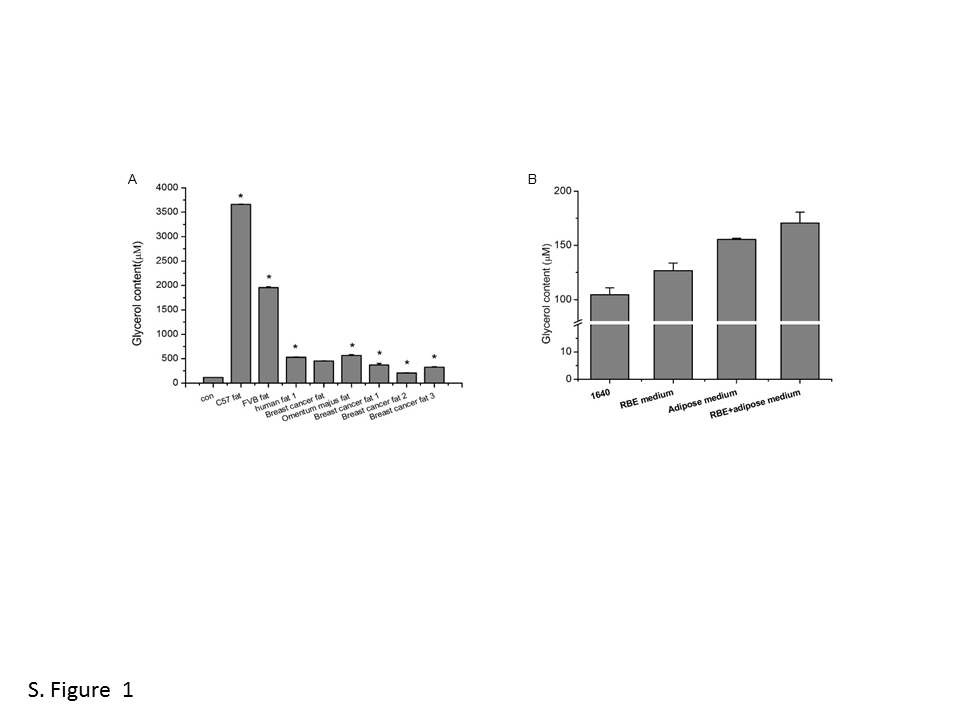
**

**Supplemental Fig. S1. Glycerol colorimetric assay.** Human cholangiocarcinoma (CCA) cell line RBE was incubated in complete growth medium or adipose tissue extracts (80 mg/mL) supplemented medium for 24 h. The culture medium were collected prior to or after cell culture for extracellular glycerol detection using colorimetric assays, with the data presented as the absorbance at 490nm (A,B). All the samples were prepared in triplicate, and all experiments were repeated at least three times. *P < 0.05.


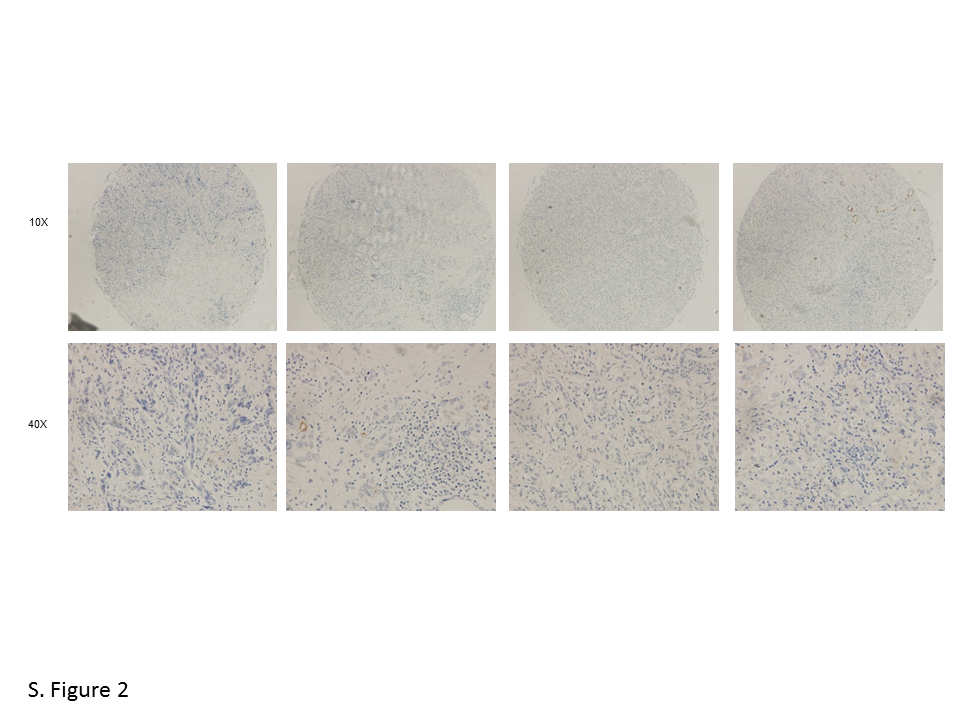


**Supplemental Fig. S2. Immunohistochemistry staining of FABP4 expression in cholangiocarcinoma tissue array.** Human CCA tissue microarray was obtained from Outdo Biotech Co., Ltd. (Shanghai, China). The FABP4 antibody (EPR3579, Abcam, MA, USA, 1:200) was used for IHC staining.


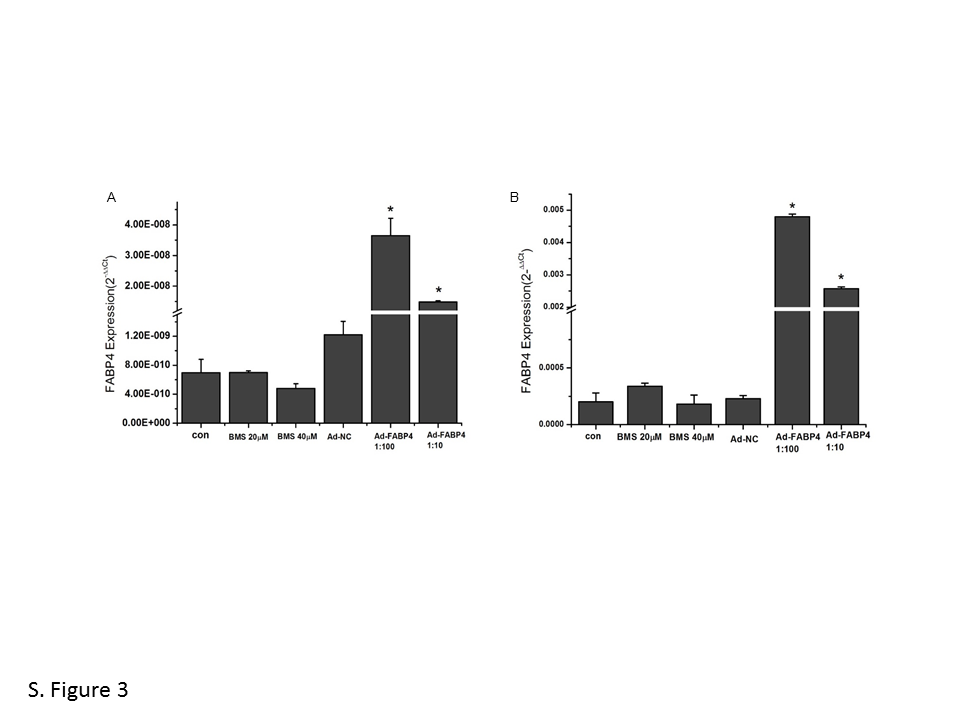


**Supplemental Fig. S3. Real time PCR detection of FABP4 mRNA expression.** Human CCA cell line RBE (A) and Hccc-9810 (B) were treated with BMS309403 (20 μg/mL or 40 μg/mL) for 24 h, as the same time these cell lines were infected with recombinant FABP4 adenovirus or control virus (1:10, 1:100), and mRNA expression of FABP4 was determined and are expressed relative to β-actin. All the samples were prepared in triplicate, and all experiments were repeated at least three times. *P < 0.05.


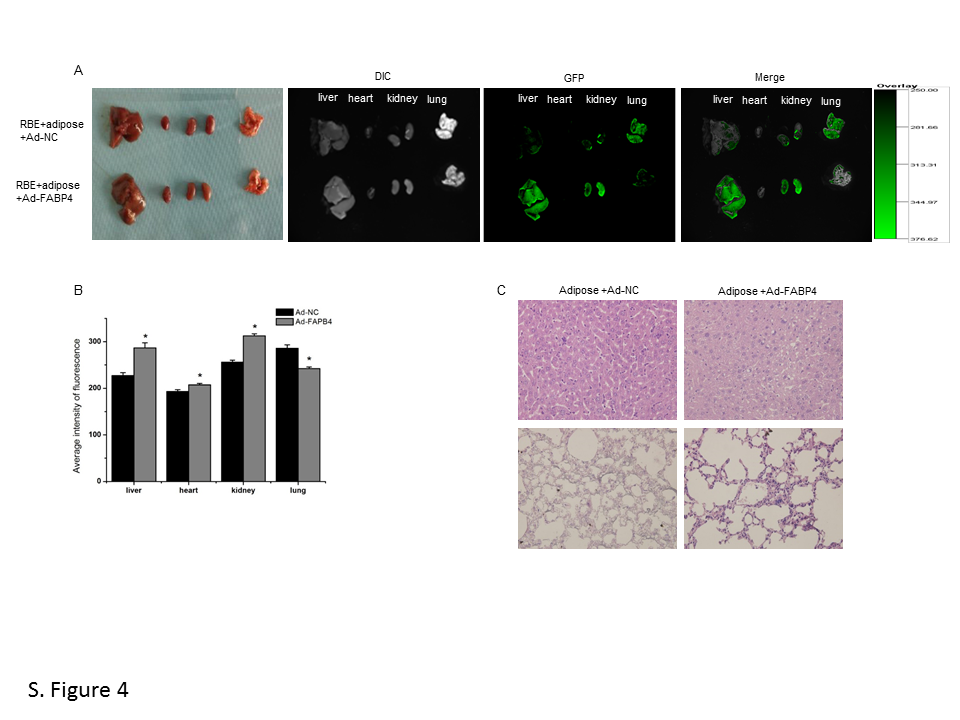


**Supplemental Fig. S4. FABP4 overexpression affects CCA metastasis in vivo.** 1×10^6^ RBE cells were infected with recombinant FABP4 adenovirus or control virus (1:10, 1:100) for 24 h, and incubated in adipose extracts medium for additional 24 h prior to tail vein injection of Balb/C nude mice. The in vivo migration and invasion of RBE cells were observed under the living animal imaging system 24 h after inoculation (A), and were quantified by fluorescence intensity (B). The mice were euthanized one month after injection. The livers and lungs were fixed in formaldehyde, embedded by paraffin, made into 4 μm sections, and stained using H&E assay (C). n = 5 per group, *P < 0.05.


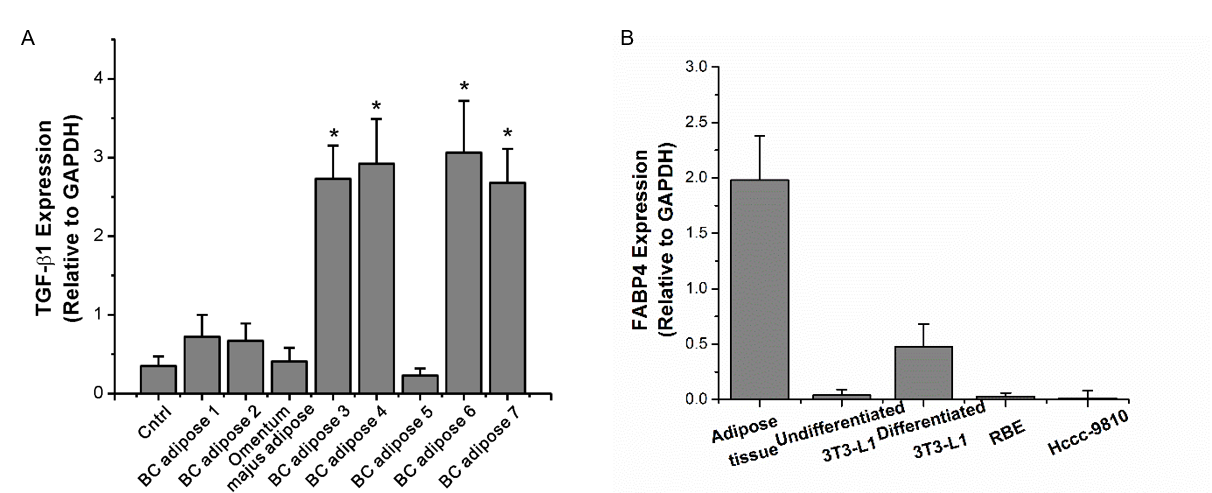


**Supplemental Fig. 5. Quantification of Western blot.** The protein expression of TGF-β of RBE cells cocultured with different adipose tissues(A), and FABP4 in different samples (B) was quantified using Image J. The band densitometry analysis was carried out relative to the loading control β-actin. *P < 0.05.


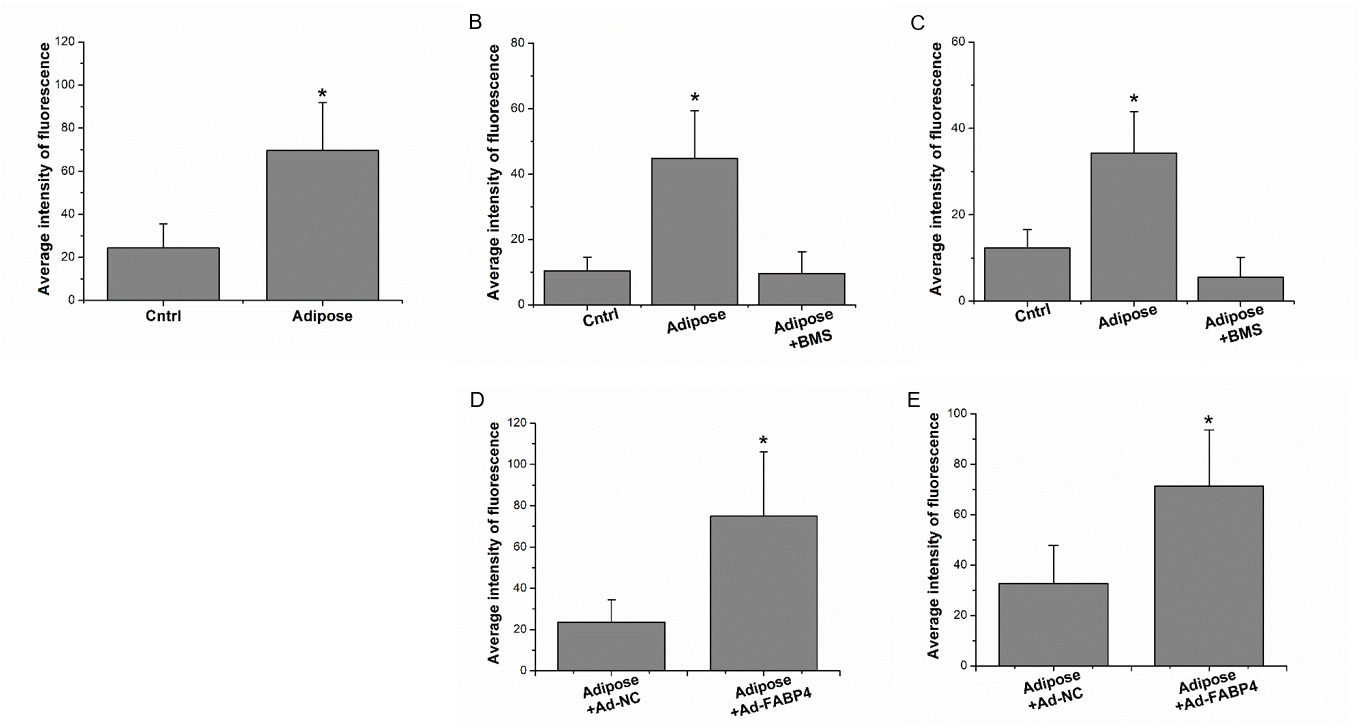


**Supplemental Fig. 6. Quantification of Bodipy staining.** The intracellular lipids in adipose cocultured RBE cells (A), BMS309403 treated RBE cells (B), BMS309403 treated Hccc-9810 cells (C), FABP4 over-expressed RBE cells (D), and FABP4 over-expressed Hccc-9810 cells (E) were visualized by the fluorescence dye Bodipy (20 μg/mL), followed by fluorescence intensity analysis using Image J. All the samples were prepared in triplicate, and all experiments were repeated at least three times. *P < 0.05.
